# Effects of Hurricane Michael on Access to Care for Pregnant Women and Associated Pregnancy Outcomes

**DOI:** 10.3390/ijerph18020390

**Published:** 2021-01-06

**Authors:** Ke Pan, Leslie Beitsch, Elaina Gonsoroski, Samendra P. Sherchan, Christopher K. Uejio, Maureen Y. Lichtveld, Emily W. Harville

**Affiliations:** 1 Department of Epidemiology, School of Public Health and Tropical Medicine, Tulane University, New Orleans, LA 70112, USA; eharvill@tulane.edu; 2 Department of Behavioral Sciences and Social Medicine, College of Medicine, Florida State University, Tallahassee, FL 32306, USA; les.beitsch@med.fsu.edu; 3 Department of Geography, College of Social Sciences and Public Policy, Florida State University, Tallahassee, FL 32306, USA; edg17@my.fsu.edu (E.G.); cuejio@fsu.edu (C.K.U.); 4 Department of Environmental Health Sciences, School of Public Health and Tropical Medicine, Tulane University, New Orleans, LA 70112, USA; sshercha@tulane.edu (S.P.S.); mlichtve@tulane.edu (M.Y.L.)

**Keywords:** disaster, prenatal care, perinatal outcomes

## Abstract

*Background*: Disasters are associated with worse perinatal outcomes, perhaps due to inadequate prenatal care (PNC). *Methods*: Using 2017–2019 Florida vital statistics, we compared PNC use before and after Hurricane Michael. We categorized counties as most affected (Area A) or less affected (Area B and C). We examined whether Michael’s effects on perinatal outcomes varied by maternity care availability and used the Baron and Kenny method to assess whether delayed PNC initiation mediated perinatal outcomes. Log-binomial regression and semi-parametric linear regression were used, controlling for maternal and ZIP code tabulation area characteristics. *Results*: Compared to the one-year period pre-Michael, the week of the first PNC was later in all areas in the one-year period post-Michael, with the largest change in Area A (adjusted difference 0.112, 95% CI: 0.055–0.169), where women were less likely to receive PNC overall (aRR = 0.994, 95% CI = 0.990–0.998) and more likely to have inadequate PNC (aRR = 1.193, 95% CI = 1.127–1.264). Michael’s effects on perinatal outcomes did not vary significantly by maternity care availability within Area A. Delayed PNC initiation appeared to mediate an increased risk in small for gestational age (SGA) births after Michael. *Conclusion*: Women in Area A initiated PNC later and had a higher likelihood of inadequate PNC. Delayed PNC initiation may partially explain increased risk of SGA.

## 1. Introduction

Disaster can produce temporary or permanent “maternity care deserts”, as the March of Dimes terms areas with no delivery or prenatal care [1]. While health care is clearly necessary for birth emergencies, limited access to obstetric care has also been associated with worse birth outcomes and maternal health [2,3,4]. Studies postdisaster, unsurprisingly, tend to find an increase in the proportion of women receiving late and often inadequate prenatal care (PNC) [5]. However, a few studies have found otherwise [6,7,8]. Other studies implicitly or explicitly consider lack of PNC to be one mediator of disaster effects. For instance, Zahran et al. found an increase in fetal distress after Hurricane Andrew and recommended more and better postdisaster PNC as a mitigating strategy [9]. 

The effect of lapses in PNC after disaster is related to individual risk factors and social determinants of health. Some studies indicate that the number of PNC visits can be reduced without significant harm to maternal or infant health [10]. The selection factors that cause a woman to enter PNC early and/or be treated frequently are strongly associated with outcomes that are both positive (women who are health-conscious and planning a pregnancy will enter care early) and negative (women with complications will be seen more often) [11]. Currie et al. examined the relationship between hurricanes and birth outcomes, and found that, while exposure to a hurricane was associated with a reduced likelihood of having at least seven prenatal visits and of having adequate PNC, lack of PNC was not a mediating factor between hurricane exposure and low birth weight or abnormal newborn conditions [12]. Other studies with less formal considerations of the effect of PNC on postdisaster pregnancy outcomes control for adequacy of PNC in their analysis, usually with little effect [6,7,13,14]. 

In addition, while birth outcomes like infant mortality, low birth weight, and preterm birth (PTB) are strong indicators of perinatal health, other important yet overlooked peripartum outcomes may be affected by health care availability. A study of women affected by the Fukushima earthquake and nuclear disaster found that discontinuation of scheduled PNC or switching to another medical institution for PNC was associated with a reduced likelihood of breastfeeding [15]. Caesarean section rates were found to be higher after Hurricane Katrina [5] and after an earthquake in Azerbaijan [16]. PNC also has benefits beyond the strictly medical. The GUMBO study of post-Katrina New Orleans found that women who participated in Healthy Start reported more mental health support and prenatal education than women in traditional PNC and had similar birth outcomes, despite having a less favorable risk profile [17]. Mothers who attended an intervention in post-earthquake Nepal were more likely to know danger signs for pregnancy complications and to have institutional deliveries [18].

Even without disaster, many women in rural areas have challenges accessing care, with longer distances to health care services and limited availability of health care providers [1]. In the past decades, the number of hospitals providing obstetric services and the number of maternity care providers have been declining in rural areas, which leads to access inequities for PNC [1]. Pregnant women in rural areas are less likely to initiate PNC in their first trimesters [19] and are more likely to have pregnancy-related morbidity and mortality compared to pregnant women in nonrural areas in the U.S. [20]. In October 2018, Hurricane Michael, a category 5 hurricane targeting the largely rural Florida Panhandle, caused over 400,000 power outages, damaged state roads, required 44,750 shelter stays, and downed over 40,000 communication lines [21]. As a consequence, several health care facilities experienced temporary or permanent closure.

In this study, we examine the effects of a major hurricane (category 5) targeting a largely rural region, with respect to availability of PNC, timing of initiation of PNC, adequacy of PNC, and their effects on perinatal outcomes. We hypothesized that adverse birth outcomes and caesarean section rates would rise in the aftermath of the disaster, that breastfeeding would decline, and that these changes would be partially mediated by availability of and access to PNC and obstetric care.

## 2. Methods

### 2.1. Data Source

Vital statistics data for 2017–2019 were obtained from the State of Florida. Births were categorized as occurring in the year before and after the date of Hurricane Michael (before: 6 October 2017–6 October 2018, after: 7 October 2018–7 October 2019). 

### 2.2. Affected Areas

Based on FEMA disaster declarations [22], Florida counties were divided into 3 categories reflecting the extent of impact from Hurricane Michael: counties receiving both public and individual assistance (Area A), counties receiving only public assistance (Area B), and counties receiving neither public nor individual assistance (Area C). Individual assistance is provided to individuals who have sustained losses, while public assistance funds are allocated to repair or reconstruct public facilities or infrastructure. Individual assistance is only available in the most affected areas.

### 2.3. Health Care Availability

*Nonhospital care providers:* based on the 2019 AtoZdatabases Healthcare Professionals database [23], a list of clinics, doctor’s offices, and care providers (including physicians, allopathic and osteopathic physicians, midwives, nurse practitioners, nurses) that potentially treated pregnant women or neonates was compiled. The specific categories that were used to search can be found in the Supplementary File. This produced a list of 760 providers, 429 of which were nonduplicates. Health facilities were contacted by phone and asked if they treated pregnant women, if they had closed due to the hurricane, and if so, for how long. In addition, we conferred with local health care and public health officials to determine whether any clinics had closed permanently in the aftermath of the storm and thus were missing from the list.

*Hospitals:* The Agency for Health Care Administration (AHCA) for the State of Florida provided a list of hospitals potentially affected by the hurricane, and the dates of evacuations, closings, and reopenings. Excluded from our analysis are facilities that are unlikely to provide obstetrical care, such as rehabilitation, long-term care, and substance abuse facilities.

Availability of maternity care in a ZIP code on a given day was therefore based on (a) whether a delivery hospital was open, and (b) the number of prenatal clinics open. For each delivery, we categorized the availability of healthcare into 3 levels based upon the date of the delivery: in a ZIP code with open delivery hospital(s) and prenatal clinic(s); in a ZIP code with either an open delivery hospital or prenatal clinic but not both; in a ZIP code with neither of these. We limited analysis of maternity care to the most affected area of the state, where residents were eligible for individual FEMA aid (Area A). Births were categorized based on mother’s residence, whether the mother resided in a ZIP code with both a delivery hospital and a clinic, only a clinic, or neither. If a hospital or clinic closed temporarily due to the hurricane, the birth was categorized based on availability when it occurred. 

### 2.4. Outcomes 

The periods between 6 October 2017–6 October 2018 and those during 7 October 2018–7 October 2019 were compared. Access to PNC services before and after Hurricane Michael were evaluated by whether pregnant women had any PNC visits before delivery, the month of first PNC visit, and the Kotelchuck Index. There are four adequacy categories in the Kotelchuck Index: adequate plus, adequate, intermediate, and inadequate [24]. We further grouped the Kotelchuck Index into two levels: adequate plus/adequate and intermediate/inadequate for mediation analysis.

The birth outcomes examined included incidence of PTB, low birth weight, and small for gestational age (SGA). PTB was defined as a birth before 37 weeks of gestation. The algorithm reported by Klebanoff et al. was used to distinguish spontaneous vs. indicated preterm births [25]. Indicators of spontaneous PTB included premature rupture of membranes, labor characteristics (i.e., prolonged labor, precipitous labor, attempted forceps, attempted vacuum, augmentation, and trial of labor), and vaginal delivery, while induction and caesarean section were associated with indicated PTB. Using the Ohio birth certificates from 2006 to 2012, the kappa statistic of the algorithm was 0.68 (95% CI 0.52, 0.83); predictive values for spontaneous and indicated onset were 85% (95% CI 75%, 92%) and 89% (95% CI 71%, 98%) respectively. Low birth weight was defined as a birthweight of an infant of 2500 g or less, regardless of gestational age. SGA was defined by birthweight below the 10th percentile for gestational age based on national standard [26]. Mode of delivery was defined as caesarean section vs. other methods. Breastfeeding was based on the indicator for infant being breastfed between birth and discharge.

### 2.5. Covariates

Confounders were chosen from known risk factors and included in models based on whether the distribution of the factors were different before and after Hurricane Michael. Variables examined as potential confounders were maternal age, race, ethnicity, smoking during pregnancy, alcohol use, education, prepregnancy BMI and enrollment in the U.S. Department of Agriculture’s Supplemental Nutrition Program for Women, Infants, and Children (WIC) program. Covariate missing data was minimal: maternal age (0.0025%), education (0.97%), race/ethnicity (1.28%), prepregnancy BMI (5.63%), WIC enrollment (1.24%), smoking during pregnancy (0.38%), and alcohol drinking during pregnancy (1.51%), so complete case analysis was conducted. 

To control for sociodemographic and geographic differences between ZIP code areas, we obtained the ZIP Code Tabulation Area (ZCTA) level data from American Community Survey (2018 5-year estimates) for the total population, the percentage of the population with health insurance, the percentage of population living below the poverty level, the percentage of white alone population, and percentage of Hispanic or Latino population. We additionally controlled for the percentage of the population living in an urban area (classifications defined by the 2010 Census Urban and Rural Classification and Urban Area Criteria) [27]. The ZCTA is similar but not exactly identical to postal ZIP codes. ZCTAs occasionally differ from postal ZIP codes for reasons such as physical barriers (water bodies), large postal receiving centers, post office box addresses, and less frequently updated geographic boundaries [28]. Therefore, we further linked the ZCTAs code to ZIP code using the 2018 ZIP code to ZCTA crosswalk table provided by the Uniform Data System Mapper [29].

### 2.6. Statistical Analysis

Models were created to examine whether outcomes differed by area, time period, and whether the area was a maternity care desert or not. 

First, we examined the effect of Hurricane Michael on PNC receipt and association between PNC and WIC receipt. A semi-parametric linear model was used to model time of first PNC visit comparing the periods before and after the hurricane. Log-binomial regression was used for binary outcomes. The linearity assumption was checked using linear graphs between predictor variables and predicted log probabilities and no violation was found. Collinearity assumptions were checked using the variance inflation factor (VIF). All VIFs were less than 2, which indicates the stability of the regression coefficients. All the estimates were compared unadjusted and after adjusting for potential confounders. 

Next, we examined whether the effects of the hurricane on perinatal outcomes varied by whether the area was a maternity care desert, adding interaction terms to the models comparing pre- and posthurricane outcomes (overall effects had been previously examined [30]). In this analysis, generalized estimating equations (GEE) with exchangeable working correlation were used to control for the correlation between births from the same ZIP code area. We also conducted a sensitivity analysis assuming that all clinics that did not answer our call/questions provided PNC and did not close during the hurricane.

Finally, we examined how care receipt might have affected perinatal outcomes. We looked at whether the relationship between hurricane exposure and outcomes was mediated by delayed PNC initiation. Because we did not detect interaction between hurricane exposure and gestational month of PNC initiation, we adopted the Baron and Kenny method [31]: (a) test whether exposure to the hurricane was associated with adverse outcomes; (b) test whether hurricane exposure was associated with delayed PNC initiation; (c) test whether delayed PNC initiation use was associated with adverse outcomes, controlling for hurricane exposure. If all three criteria were met and the regression coefficients were both positive in (b) and (c), the Sobel test was used to formally test whether the indirect effect was statistically significant [32]. (d) Lastly, we assessed whether the association between hurricane exposure and adverse outcome shrank after adjusting for delayed PNC initiation.

Statistical analyses were performed using the software SAS 9.4 (SAS Inc., Cary, NC, USA). 

This analysis was approved by the Institutional Review Boards of Tulane University (2019-529-TUHSC), Florida State University, and the Florida Department of Health.

## 3. Results

The study population is described in Table 1. In the most affected area, the educational and race distribution of the population giving birth changed slightly, with a lower proportion in the associates/some college education group after Hurricane Michael. A lower proportion of women received WIC benefits and a higher proportion of women smoked during pregnancy after the storm. The proportion of women receiving WIC benefits after Michael remained significantly decreased after adjusting for maternal education, race/ethnicity, and age in all areas and the greatest decline in area A (probability of receiving WIC benefits: area A: aRR = 0.906, 95% CI: 0.879, 0.934; area B: aRR = 0.944, 95% CI: 0.900, 0.989; area C: aRR = 0.949, 95% CI: 0.943, 0.955). Moreover, having adequate/adequate plus PNC was associated with a slightly higher probability of receiving WIC benefits after adjusting for maternal age, race/ethnicity, and education (area A: aRR = 1.046, 95% CI: 1.017, 1.075; area C: aRR = 1.019, 95% CI: 1.012, 1.025). 

### 3.1. Hurricane and PNC Use

The gestational month of the first prenatal visit was later in all three areas using the linear regression models with control for clustering (GEE), but the largest change was in the most affected area (adjusted difference in gestational month: 0.112, 95% CI: 0.055, 0.169), (Table 2). In area A, log-binomial regression models show that women were less likely to receive PNC overall (aRR = 0.994, 95% CI = 0.990, 0.998), and more likely to have received inadequate PNC (inadequate/intermediate) (aRR = 1.193, 95% CI = 1.127, 1.264). 

### 3.2. Hurricane Michael and Maternity Care Availability

**Figure 1:**
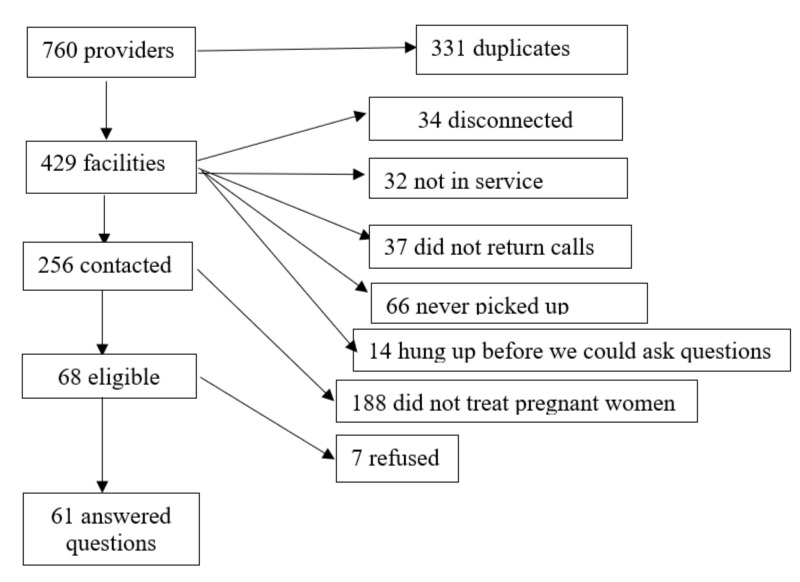
Results of phone calls to health facilities.

Of the 429 health facilities, 188 did not treat pregnant women. We approached 68 facilities (Figure 1 provides a flowchart for disposition of other facilities). Seven facilities refused to answer questions; of the remaining 61 facilities that responded, 25 facilities closed during Michael (range: 1 day–2 weeks except one facility that closed permanently after Michael), and only three of them reported referring pregnant women seeking healthcare to other health facilities. The remainder were closed, hung up, did not return calls, or did not answer the phone. Five hospitals that provide obstetrical services reported damage during Michael, 2 with major damage.

### 3.3. Hurricane Michael and Perinatal Outcomes by Maternity Care Availability

GEE models with exchangeable working correlation matrix showed that the effects of the hurricane on all outcomes of interest were not significantly different across maternity care availability within area A (Table 3). Among the perinatal outcomes of interest, only low birth weight was in the direction of the hypothesis. There was no effect of hurricane on low birth weight in the areas with good access (aRR = 1.102, 95% CI: 0.970, 1.252), and a stronger effect in the areas with no access (aRR = 1.218, 95% CI: 1.054, 1.406); however, those effects were not statistically different (p = 0.615). In the sensitivity analysis, we assumed that all clinics that did not answer our calls/questions continued to provide PNC and did not close during hurricane Michael; results were similar (Table S1). 

### 3.4. Mediation Analysis of PNC Use

Overall, inadequate PNC (inadequate/intermediate PNC) was associated with higher risk of low birth weight, spontaneous preterm birth, induced preterm birth, and SGA compared with adequate PNC use in all areas (Table 4). Those associations were not significantly different before and after Hurricane Michael (no interaction between PNC use and hurricane). Compared to adequate PNC use, women with inadequate PNC use were more likely to have cesarean sections in area B before the hurricane, and women with inadequate PNC use were less likely to adopt breastfeeding in all areas except in Area B before the hurricane.

In the most affected area (area A), Hurricane Michael was significantly associated with low birth weight and SGA regardless of the time of PNC initiation (Table 5). Adjustment for delayed PNC initiation had very small effect: the relative risk of low birth weight shifted from 1.188 (95% CI: 1.059, 1.334) to 1.194 (95% CI: 1.064, 1.340) after further adjusting for delayed PNC initiation, while the relative risk of SGA shifted from 1.130 (95% CI: 1.031, 1.238) to 1.126 (95% CI: 1.028, 1.234) (Table 5). Although adjusting for PNC initiation did not change the association between the hurricane and SGA substantially, we identified positive associations between the hurricane and SGA, the hurricane and delayed PNC initiation, and delayed PNC initiation and SGA, adjusting for the hurricane (*p*-value for Sobel test = 0.0002). 

## 4. Discussion

In this analysis, we examined the effects of Hurricane Michael on access to care in the largely rural Florida Panhandle, and the relationships this had with perinatal outcomes. Few previous studies have formally addressed this question, particularly among rural populations, largely limiting themselves to adjusting models of birth outcomes for receipt of PNC [6,7,13,14]. We found that women living in the areas most affected by the hurricane had a later week of first prenatal visit and were less likely to receive adequate prenatal care. Moreover, delayed PNC initiation might partially mediate the association between hurricane and SGA.

That disasters would reduce access to PNC (due to either facility closures or patients’ inability to reach facilities) seems a logical assumption, but it has not always been confirmed in previous studies. While both this study and studies of Hurricane Katrina found a strong negative effect on PNC [5], a study of 9/11 found a slight increase in the proportion of women who had first trimester PNC in New York City [6] as did a study of flooding in North Dakota [7]. A study of El Niño-related floods in Ecuador found no differences in PNC initiation or number of visits [8]. There are many possible reasons for the inconsistency: type, severity and duration of the disaster; the natural environment; local infrastructure and socio-economic situation of the affected area; and people’s experience with such disasters [33]. For example, natural disasters could possibly have different impacts on the PNC use in urban and rural settings, from both the provider and the patient side. Hurricane Michael hit a largely rural region in the Florida Panhandle, which caused several hospital and clinics to close temporarily or permanently. In the most affected area, pregnant women were less likely to receive PNC or adequate PNC and started PNC later. The implications of reduction in obstetrical care may be different in urban and rural areas: in a rural area, the closure of a clinic or a hospital may eliminate the only provider in an area and women might have limited options to evacuate or schedule PNC appointments in other areas during natural disaster. However, rural women may also be more accustomed to travelling long distances to access care and may perceive less need for care to be located nearby. Rural counties that have lost obstetric care for non-disaster-related reasons had an increase in preterm births, and as would be expected, an increase in out-of-hospital births and births in hospitals without obstetrical units [2]. Obstetric unit closures in Philadelphia were also associated with worse obstetric outcomes, but this ameliorated after 2–3 years [3].

Currently there is no specific standard or guideline for providing routine PNC during and after disaster. Two disaster planning standards or guidelines for obstetric services published by the American College of Obstetricians and Gynecologists (ACOG) [34] and Stanford Medicine [35] focus on maternity instead of routine PNC. While delayed PNC might not significantly increase adverse perinatal outcomes, pregnant women with chronic diseases and pregnancy complications are more vulnerable to PNC disruptions caused by disasters. In 2012, Sphere, a global humanitarian preparedness agency, provided a set of humanitarian standards to address the needs of people with chronic conditions in disasters. It recommended documenting key chronic disease burdens to highlight health gaps that need to be addressed, maintaining treatment for chronic diseases, and avoiding sudden discontinuation of treatment, identifying and facilitating referral options, where relevant services for chronic diseases are provided [36]. These recommendations can be applied to providing routine PNC during disaster. Many PNC care providers have limited knowledge about preparedness, while many first responders have limited knowledge about PNC. Training both workforces may help address this gap [33]. In this study, among the 25 health facilities that self-reported closure during or after Michael, only three health facilities said they referred pregnant women to other health facilities before the disaster and another two health facilities claimed that there was nowhere to refer pregnant women (both in the most affected area). Increasing collaboration among physicians, insurers, health plans, pre-, during, and postdisaster may help identify high-risk women who need referrals to other PNC clinics and facilitate the referral process [33].

We did not find evidence that the effect of Hurricane Michael on adverse perinatal outcomes varied according to health care availability in the most affected area. This could be due to imperfect records of clinics that provide PNC care regarding whether they closed during Hurricane Michael. Some clinics refused to answer our questions after answering the phone; others could not be reached by phone. It is possible that those unreached clinics provided PNC, but a sensitivity analysis where those clinics were classified as providing care found no difference. 

Although we found evidence that PNC was disrupted by Hurricane Michael, the increase in adverse perinatal outcomes was not explained by delayed PNC initiation overall, which is consistent with previous studies [6,7,12,13,14]. For SGA, adjusting for PNC initiation time only shrinks the association between hurricane and SGA by 0.35%. This might be because natural disasters like hurricanes can cause adverse perinatal morbidity not only through reduced healthcare access, but also via underlying social determinants of health. According to the framework for assessing mortality and morbidity after large-scale disasters suggested by National Academies of Sciences, Engineering, and Medicine, besides infrastructure destruction, social determinants of health including environmental, social/psychosocial, human and cultural, financial, and political factors should be considered when assessing disaster-related mortality and morbidity [37]. For example, hurricanes can increase the risk of adverse perinatal outcomes by causing environmental exposure, worsened stress and mental health, physical trauma, and behavioral changes [38]. The effects of all those risk factors on perinatal outcomes also depend on the individual’s wealth and government support policies. Individual-level care is unlikely to eliminate disparities caused at the macro socioeconomic level [39]. Therefore, increasing traditional PNC use might have only a small effect on improving perinatal outcomes. 

However, PNC visits could provide an opportunity for pregnant women to receive mental health support, education in environmental health, and linkage with community resources, including nutrition and social service programs, after natural disasters. For instance, the Women, Infants and Children program (WIC), a supplemental nutrition program supported by the US Department of Agriculture, provides health care referrals and nutritional support to low-income pregnant women, postpartum women, and children up to age 5 [40]. Eligible participants referred by PNC providers to the WIC program will receive nutritious foods and health education regarding food nutrition/safety and substance use after disaster [40,41]. Moreover, the WIC program helps identify women with or at risk of depression and link them to appropriate health care services [42], which can help address the increased stress and mental health issues after disaster and improve pregnancy outcomes. After Hurricane Michael, women receiving adequate/adequate plus PNC had a slightly higher probability of receiving WIC benefits after adjusting for maternal age, race/ethnicity, and education, and the association was the strongest in area A. Similarly, following Hurricane Katrina in New Orleans, the Healthy Start program enrolled pregnant women with relatively low socio-economic status and provided social services, education, and referrals in addition to traditional PNC services. Compared to women who only received traditional PNC, pregnant women enrolled in the Healthy Start program had similar birth outcomes despite being higher risk (reporting more negative hurricane experience occurrences and more depression and PTSD symptoms) [17]. Moreover, access to WIC services can be one of the indicators of the impact of disasters on the needs of target populations [43]. WIC participation is associated with improved pregnancy outcomes, including a decrease in low birth weight, neonatal mortality, and an increase in gestational age [44,45]. Routine WIC service operations were interrupted by Superstorm Sandy in New York State [46]. Similarly, after Hurricane Michael, the proportion of pregnant women enrolled in the WIC program dropped from 50.98% to 46.51% (*p* < 0.0001) in the most affected areas. Improving partner communication among WIC provider sites, regional health offices and the state health department, and PNC providers prior to and in the aftermath may help improve PNC use and WIC enrollment after a disaster [46]. 

Strengths of this study include the large sample size and examination of multiple adverse perinatal outcomes. There are also several limitations of this study. First, we used the measurement of access to care at a ZIP code level rather than the measures specific to an individual woman. Therefore, we do not know how many women were forced to find new sources of care. Second, we did not address maternal mortality or severe maternal morbidity, as such complications are relatively rare and the area under study was sparsely populated. However, such conditions might be more directly affected by lack of care [4]. Third, as mentioned above, identification of prenatal care clinics was incomplete, as many potential locations refused to answer questions or did not answer the telephone. Fourth, this study relied on vital statistics, defining exposure by county-level damage and timing, which might not be accurate for a woman’s individual exposure. However, this tends to cause nondifferential bias because the categorization of exposure and timing is unlikely to vary by outcome. Fifth, this study lacks information on stress and mental health, behavioral change, or social mechanisms of effect. These aspects should be addressed in future studies to provide more evidence to policy makers and healthcare providers for preventing adverse perinatal outcomes after natural disaster. Lastly, the findings of this study might not apply to other countries with a different health care system to the U.S.

## 5. Conclusions

In this study, we found that women living in rural areas most strongly hit by a hurricane had a later week of first prenatal visit and were more likely to receive less than adequate prenatal care. However, the increase in adverse perinatal outcomes was not explained by delayed PNC initiation, except for SGA. More research is needed to determine the role of PNC in presenting an opportunity to provide pregnant women with support on mental health, education on environmental health, and linkage with community resources after natural disaster.

## Figures and Tables

**Table 1:** Characteristics of study population in Florida one year before and after Hurricane Michael (6 October 2017—7 October 2019).

	FEMA Individual ^a^ (Area A)			FEMA Public (Area B)			Not Affected (Area C)		
	Before	After	*p*-Value	Before	After	*p*-Value	Before	After	*p*-Value
	Mean (SE)/N (%)			Mean (SE)/N (%)			Mean (SE)/N (%)		
Total number of births	7555	7261	0.492	4334	4406	0.213	207014	206299	0.417
Maternal age	27.73 (5.72)	27.86 (5.75)	0.157	27.92 (5.89)	28.19 (5.63)	0.024	29.18 (5.83)	29.33 (5.85)	<0.0001
Maternal education level									
High School or GED or less	3173 (42.67%)	3109 (43.83%)	0.001	1858 (43.35%)	1980 (45.30%)	0.015	86579 (42.26%)	86503 (42.27%)	<0.0001
Some College Credit, but no Degree or Associate Degree	2370 (31.87%)	2069 (29.17%)		1412 (32.94%)	1314 (30.06%)		59307 (28.95%)	57797 (28.24%)	
Bachelor’s Degree and above	1893 (25.46%)	1915 (27.00%)		1016 (23.71%)	1077 (24.64%)		58962 (28.78%)	60360 (29.49%)	
Maternal ethnicity									
Non-Hispanic white	4455 (59.55%)	4191 (58.51%)	0.003	2955 (68.29%)	2925 (66.64%)	0.298	83808 (41.16%)	83183 (40.71%)	<0.0001
Hispanic white	387 (5.17%)	450 (6.28%)		498 (11.51%)	526 (11.98%)		61018 (29.97%)	62365 (30.52%)	
Black	2206 (29.49%)	2169 (30.28%)		552 (12.76%)	613 (13.97%)		45250 (22.22%)	44801 (21.92%)	
Other	433 (5.79%)	353 (4.93%)		323 (7.44%)	325 (7.40%)		13526 (6.64%)	13998 (6.85%)	
WIC program	3772 (50.98%)	3346 (46.51%)	<0.0001	1762 (41.62%)	1735 (39.54%)	0.050	91147 (44.50%)	85758 (42.14%)	<0.0001
Prepregnancy BMI	27.81 (7.31)	27.86 (7.38)	0.702	27.07 (6.76)	27.32 (7.02)	0.096	26.76 (6.50)	26.95 (6.60)	<0.0001
Smoking during pregnancy	534 (7.10%)	597 (8.28%)	0.007	445 (10.29%)	506 11.50%)	0.070	8126 (3.94%)	7755 (3.77%)	0.004
PNC started month	2.76 (1.58)	2.91 (1.72)	<0.0001	2.56 (1.55)	2.65 (1.59)	0.006	2.66 (1.68)	2.69 (1.70)	<0.0001
Whether received PNC	7423 (98.84%)	7097 (97.98%)	<0.0001	4263 (98.70%)	4336 (98.55%)	0.527	201876 (98.11%)	200220 (98.09%)	0.629
Kotelchuck Index									
Inadequate	918 (14.14%)	1202 (18.60%)	<0.0001	460 (13.10%)	562 (15.05%)	0.001	29990 (16.57%)	30309 (16.95%)	0.001
Intermediate	656 (10.11%)	714 (11.05%)		343 (9.77%)	431 (11.54%)		22696 (12.54%)	21812 (12.20%)	
Adequate	2938 (45.26%)	2847 (44.06%)		1518 (43.22%)	1463 (39.18%)		74243 (41.01%)	73336 (41.00%)	
Adequate plus	1979 (30.49%)	1699 (26.29%)		1191 (33.91%)	1278 (34.23%)		54109 (29.89%)	53399 (29.86%)	

^a^ Area A = FEMA individual; Area B = FEMA public; Area C = nonaffected; FEMA, Federal Emergency Management Agency; SE, standard error; GED, general educational development; BMI, body mass index; PNC, prenatal care.

**Table 2:** Changes in prenatal care use before after Hurricane Michael among different areas.

		Univariate Model			Multivariable Model *		
		Area A ^&^	Area B ^&^	Area C ^&^	Area A ^&^	Area B ^&^	Area C ^&^
Whether received PNC services	N	14,753	8719	409,899	14,753	8719	409,899
	RR^@^ (95% CI)	0.992 (0.988, 0.997)	0.998 (0.993, 1.003)	1.000 (0.999, 1.001)	0.994 (0.990, 0.998)	0.999 (0.994, 1.004)	1.000 (0.999, 1.001)
	*p*-value ^#^	0.001	0.382	-	0.003	0.652	-
Gestational month of first PNC	N	12,222	7306	355,541	12,222	7306	355,541
	Difference ^@^ (95% CI)	0.107 (0.050, 0.165)	0.094 (0.023, 0.165)	0.027 (0.016, 0.039)	0.112 (0.055, 0.169)	0.088 (0.017, 0.159)	0.034 (0.023, 0.045)
	*p*-value ^#^	0.007	0.071	-	0.008	0.139	-
Inadequate PNC ^^^	N	12,953	7246	359,894	12,953	7246	359,894
	RR^@^ (95% CI)	1.215 (1.145, 1.288)	1.180 (1.087, 1.281)	0.999 (0.989, 1.009)	1.193 (1.127, 1.264)	1.154 (1.064, 1.251)	1.0004 (0.991, 1.011)
	*p*-value ^#^	<0.0001	<0.0001	-	<0.0001	0.001	-

^&^ Area A = FEMA individual; Area B = FEMA public; Area C = nonaffected; FEMA, Federal Emergency Management Agency; ^@^ Difference: difference in gestational month; RR: cumulative risk ratio, CI: confidence interval, compared year before and after Michael. ^#^ *p*-value is to compare area A and B to area C, respectively. * Adjusting for: mother’s age, education, ethnicity, smoking during pregnancy, and whether in WIC program; ^^^ Kotelchuck Index: inadequate or intermediate PNC compared to adequate plus/adequate PNC. PNC, prenatal care.

**Table 3:** Changes in perinatal outcomes after Hurricane Michael by maternity care availability within Area A ^&^.

		Univariate Model			Multivariable Model *		
		Both Clinic and Hospital Available	Either Clinic or Hospital Available	None	Both Clinic and Hospital Available	Either Clinic or Hospital Available	None
		RR (95% CI) ^#^			RR (95% CI) ^#^		
Low birth weight (LBW)	After vs. before	1.048 (0.869, 1.263)	1.133 (0.838, 1.530)	1.234 (1.062, 1.433)	1.102 (0.970, 1.252)	1.128 (0.857, 1.485)	1.218 (1.054, 1.406)
	*p*-value for interaction	-	-	0.446	-	-	0.615
Spontaneous preterm birth (SPTB)	After vs. before	1.124 (0.813, 1.553)	1.068 (0.806, 1.414)	0.880 (0.744, 1.041)	1.253 (0.826, 1.902)	1.024 (0.777, 1.350)	0.864 (0.730, 1.024)
	*p*-value for interaction	-	-	0.314	-	-	0.208
Induced preterm birth (IPTB)	After vs. before	0.813 (0.551, 1.200)	1.218 (1.055, 1.406)	0.949 (0.759, 1.186)	0.852 (0.564, 1.287)	1.187 (1.001, 1.407)	0.932 (0.741, 1.172)
	*p*-value for interaction	-	-	0.188	-	-	0.303
Small for gestational age (SGA)	After vs. before	1.425 (1.286, 1.579)	0.979 (0.812, 1.181)	1.146 (1.012, 1.296)	1.389 (1.190, 1.621)	1.002 (0.850, 1.182)	1.121 (1.003, 1.253)
	*p*-value for interaction	-	-	0.159	-	-	0.244
Caesarean section	After vs. before	1.035 (0.883, 1.212)	1.041 (0.945, 1.147)	1.003 (0.937, 1.074)	1.056 (0.912, 1.221)	1.049 (0.951, 1.156)	0.995 (0.929, 1.066)
	*p*-value for interaction	-	-	0.807	-	-	0.620
No breast-feeding	After vs. before	1.004 (0.962, 1.047)	0.886 (0.778, 1.010)	1.029 (0.964, 1.099)	1.045 (0.961, 1.137)	0.882 (0.928, 1.080)	1.001 (0.928, 1.080)
	*p*-value for interaction	-	-	0.304	-	-	0.421

^#^ RR: cumulative risk ratio, CI: confidence interval. ^*^ LBW adjusting for: mother’s education, age, ethnicity, whether in WIC program, and each zip-code area’s total population, health insurance coverage, poverty percentage, urban/rural percentage, race and ethnicity percentage; SPTB, IPTB, SGA, C-section, breastfeeding adjusting for: mother’s age, education, ethnicity, prepregnancy BMI, whether in WIC program, and each zip-code area’s total population, health insurance coverage, poverty percentage, urban/rural percentage, race and ethnicity percentage. ^&^ Area A = FEMA individual; FEMA, Federal Emergency Management Agency.

**Table 4:** The association between Kotelchuck Index and perinatal outcomes before and after Hurricane Michael ^#^.

	Area A ^&^			Area B ^&^			Area C ^&^		
	Before	After	*p*-Value ^	Before	After	*p*-Value ^	Before	After	*p*-Value ^
	RR (95% CI) ^*^			RR (95% CI) ^*^			RR (95% CI) ^*^		
Low birth weight (LBW)									
Inadequate	2.238 (1.644, 3.046)	1.873 (1.455, 2.411)	0.809	2.659 (1.778, 3.977)	3.908 (2.672, 5.718)	0.398	1.766 (1.675, 1.862)	1.873 (1.777, 1.974)	0.228
Intermediate	1.238 (0.814, 1.884)	1.094 (0.760, 1.576)		1.893 (1.164, 3.080)	1.706 (1.027, 2.836)		1.057 (0.986, 1.134)	1.135 (1.059, 1.217)	
Adequate plus	4.026 (3.181, 5.096)	3.888 (3.169, 4.770)		3.213 (2.327, 4.437)	3.839 (2.811, 5.518)		3.045 (2.920, 3.176)	3.076 (2.948, 3.207)	
Spontaneous preterm birth (SPTB)									
Inadequate	1.245 (0.898, 1.727)	1.666 (1.223, 2.268)	0.145	1.780 (1.125, 2.815)	1.686 (1.0816, 2.631)	0.493	1.468 (1.388, 1.553)	1.563 (1.478, 1.654)	0.347
Intermediate	1.902 (1.390, 2.603)	1.718 (1.199, 2.461)		1.467 (0.859, 2.505)	1.773 (1.115, 2.820)		1.289 (1.207, 1.375)	1.378 (1.290, 1.472)	
Adequate plus	2.196 (1.741, 2.771)	2.985 (2.320, 3.842)		1.565 (1.078, 2.272)	2.174 (1.537, 3.073)		2.119 (2.025, 2.218)	2.199 (2.099, 2.303)	
Induced preterm birth (IPTB)									
Inadequate	1.259 (0.870, 1.822)	1.734 (1.257, 2.392)	0.411	1.576 (1.008, 2.466)	1.388 (0.967, 1.991)	0.119	1.353 (1.270, 1.440)	1.502 (1.414, 1.596)	0.064
Intermediate	1.475 (1.007, 2.160)	1.776 (1.222, 2.581)		2.087 (1.332, 3.271)	1.020 (0.651, 1.601)		1.253 (1.168, 1.344)	1.374 (1.282, 1.472)	
Adequate plus	2.733 (2.145, 3.454)	3.560 (2.798, 4.631)		2.971 (2.201, 4.010)	2.109 (1.623, 2.740)		2.688 (2.569, 2.812)	2.779 (2.656, 2.908)	
Small for gestational age (SGA)									
Inadequate	1.672 (1.372, 2.038)	1.374 (1.152, 1.639)	0.344	2.063 (1.505, 2.829)	2.519 (1.899, 3.341)	0.802	1.346 (1.295, 1.398)	1.361 (1.309, 1.414)	0.855
Intermediate	0.911 (0.681, 1.219)	0.994 (0.781, 1.265)		1.087 (0.699, 1.689)	1.169 (0.793, 1.723)		0.982 (0.936, 1.031)	0.962 (0.915, 1.011)	
Adequate plus	1.874 (1.592, 2.205)	1.881 (1.622, 2.182)		2.102 (1.899, 3.341)	2.196 (1.718, 2.807)		1.585 (1.535, 1.637)	1.579 (1.528, 1.631)	
Cesarean section									
Inadequate	1.044 (0.927, 1.175)	1.053 (0.950, 1.168)	0.916	1.197 (1.024, 1.399)	1.032 (0.896, 1.187)	0.061	0.950 (0.932, 0.969)	0.951 (0.933, 0.970)	0.733
Intermediate	0.966 (0.845, 1.104)	0.924 (0.812, 1.050)		1.076 (0.893, 1.295)	0.814 (0.678, 0.977)		0.982 (0.962, 1.003)	0.990 (0.969, 1.011)	
Adequate plus	1.207 (1.114, 1.307)	1.233 (1.137, 1.338)		1.254 (1.121, 1.402)	1.053 (0.948, 1.174)		1.162 (1.146, 1.179)	1.153 (1.137, 1.170)	
No breastfeeding									
Inadequate	1.140 (1.000, 1.300)	1.240 (1.099, 1.399)	0.602	1.132 (0.911, 1.406)	1.230 (1.001, 1.512)	0.190	1.380 (1.334, 1.427)	1.484 (1.437, 1.533)	<0.0001
Intermediate	1.161 (1.002, 1.346)	1.109 (0.947, 1.298)		0.993 (0.717, 1.308)	1.073 (0.833, 1.381)		1.078 (1.033, 1.126)	1.132 (1.086, 1.180)	
Adequate plus	0.941 (0.832, 1.063)	1.011 (0.890, 1.149)		0.872 (0.717, 1.060)	1.161 (0.973, 1.384)		1.281 (1.241, 1.322)	1.174 (1.138, 1.212)	

^#^ Reference group: adequate PNC care; all models are adjusted for maternal age, education, race/ethnicity, prepregnancy BMI, smoke during pregnancy, alcohol use during pregnancy, whether in WIC program; ^&^ Area A = FEMA individual; Area B = FEMA public; Area C = nonaffected; FEMA, Federal Emergency Management Agency; ^^^ *p*-value for interaction. ^*^ RR: cumulative risk ratio, CI: confidence interval.

**Table 5:** The effect of PNC on the association between Hurricane Michael and perinatal outcomes in different areas.

		Area A ^&^		Area B ^&^		Area C ^&^	
		Difference ^@^	95% CI ^@^	RD ^@^	95% CI ^@^	RD ^@^	95% CI ^@^
Hurricane and Gestational month of first PNC *	**After vs. Before**	**0.112**	0.055, 0.169	0.088	0.017, 0.159	0.034	0.023, 0.045
Low birth weight (LBW)							
		**RR ^@^**	**95% CI ^@^**	**RR ^@^**	**95% CI ^@^**	**RR ^@^**	**95% CI ^@^**
Gestational month of first PNC and ^#^		1.003	0.967, 1.041	1.049	0.999, 1.100	0.956	0.949, 0.963
Hurricane and LBW ^^^	After vs. Before	1.188	1.059, 1.334	1.025	0.875, 1.202	1.011	0.989, 1.202
Hurricane and LBW adjusting for gestational month of first PNC ^^^	After vs. Before	1.194	1.064, 1.340	1.029	0.877, 1.206	1.013	0.990, 1.036
Spontaneous preterm birth (SPTB)							
Gestational month of first PNC and SPTB ^#^		0.897	0.851, 0.945	0.881	0.812, 0.955	0.870	0.862, 0.879
Hurricane and SPTB ^^^	After vs. Before	0.895	0.779, 1.027	1.186	0.968, 1.453	1.003	0.977, 1.029
Hurricane and SPTB adjusting for gestational month of first PNC ^^^	After vs. Before	0.903	0.787, 1.037	1.198	0.978, 1.468	1.007	0.982, 1.034
Induced preterm birth (IPTB)							
Gestational month of first PNC and IPTB ^#^		0.835	0.783, 0.890	0.932	0.881, 0.985	0.879	0.871, 0.887
Hurricane and IPTB ^^^	After vs. Before	0.977	0.850, 1.123	1.072	0.916, 1.255	1.015	0.989, 1.042
Hurricane and IPTB adjusting for inadequate/intermediate PNC ^^^	After vs. Before	0.991	0.862, 1.138	1.086	0.927, 1.271	1.019	0.993, 1.046
Small for gestational age (SGA)							
Gestational month of first PNC and SGA ^#^		1.067	1.040, 1.094	1.101	1.060, 1.143	1.031	1.025, 1.036
Hurricane and SGA ^^^	After vs. Before	1.130	1.031, 1.238	1.059	0.922, 1.216	0.986	0.968, 1.005
Hurricane and SGA adjusting for Gestational month of first PNC ^^^	After vs. Before	1.126	1.028, 1.234	1.056	0.919, 1.212	0.985	0.967, 1.003
Cesarean section							
Gestational month of first PNC and Cesarean-section ^#^		0.991	0.975, 1.007	1.014	0.993, 1.036	0.976	0.973, 0.978
Hurricane and Cesarean section ^^^	After vs. Before	1.011	0.963, 1.061	0.999	0.9365 1.067	0.988	0.980, 0.997
Hurricane and Cesarean section adjusting for Gestational month of first PNC ^^^	After vs. Before	1.015	0.967, 1.065	1.001	0.938, 1.069	0.989	0.980, 0.997
No breastfeeding							
Gestational month of first PNC and no breastfeeding ^#^		1.009	0.988, 1.029	1.006	0.973, 1.040	1.021	1.016, 1.026
Hurricane and no breastfeeding ^^^	After vs. Before	0.972	0.909, 1.041	1.057	0.950, 1.177	1.051	1.033, 1.070
Hurricane and no breastfeeding adjusting for Gestational month of first PNC ^^^	After vs. Before	0.970	0.906, 1.038	1.055	0.948, 1.175	1.050	1.032, 1.069

^&^ Area A = FEMA individual; Area B = FEMA public; Area C = nonaffected; FEMA, Federal Emergency Management Agency; * adjusting for: hurricane, mother’s age, education, race/ethnicity, whether in WIC program, and smoking during pregnancy; ^#^ Adjusting for: maternal age, education, race/ethnicity, prepregnancy BMI, whether in WIC program, smoke during pregnancy, and alcohol use during pregnancy; ^^^ LBW adjusting for: mother’s age, education, race/ethnicity, whether in WIC program, and smoking during pregnancy; PTB, SPTB, IPTB, SGA, C-section, breastfeeding adjusting for: mother’s age, education, race/ethnicity, prepregnancy BMI, whether in WIC program, and smoking during pregnancy. ^@^ Difference: difference in gestational month; RR: cumulative risk ratio, CI: confidence interval.

## Data Availability

The data presented in this study are available on request from the Florida Department of Health, upon signing of data use agreement and Institutional Review Board approval.

## Supplementary Materials

The following are available online at https://www.mdpi.com/1660-4601/18/2/390/s1, Table S1: Changes in perinatal outcomes after Hurricane Michael by maternity care availability within Area A& (sensitivity analysis).
