# Supplementary material for: Effects of Hurricane Michael on Access to Care for Pregnant Women and Associated Pregnancy Outcomes
Source: Int J Environ Res Public Health. 2021 Jan 6;18(2):390. doi: 10.3390/ijerph18020390 (PMC7825524; doi:10.3390/ijerph18020390)
Supplement: Supplementary file 1 [file ijerph-18-00390-s001.pdf]

Supplementary file

The keywords that were searched in the 2019 AtoZdatabases Healthcare Professionals database: "Allopathic & Osteopathic Physicians", "Ambulatory Health Care Facilities # Clinic/Center # Medically Fragile Infants and Children Day Care", "Clinic/Center # Ambulatory Family Planning Facility", "Clinic/Center #Birthing", "General Acute Care Hospital # Rural", "Midwife", "Nurse Practitioner #Family #Neonatal #Obstetrics & Gynecology #Women's Health #Lactation Consultant #Neonatal Intensive Care", "Registered Nurse #Lactation Consultant #Neonatal Intensive Care #Women's Health Care", and "Respiratory, Rehabilitative & Restorative Service Providers # Res # Neonatal/Pediatrics".

Table S1 Changes in perinatal outcomes after Hurricane Michael by maternity care availability within Area A[&] (sensitivity analysis)

		Univariate model			Multivariate model [*]		
		Both clinic and hospital available	Either clinic or hospital available	None	Both clinic and hospital available	Either clinic or hospital available	None
		RR (95%CI)[#]			RR (95%CI)[#]		
**Low birth weight (LBW)**	**After vs before**	1.114 (0.956, 1.298)	1.214 (1.023, 1.441)	1.167 (0.942, 1.445)	1.094 (0.935, 1.280)	1.213 (1.031, 1.428)	1.138 (0.932, 1.391)
	**p-value for interaction**	-	-	0.783	-	-	0.710
**Spontaneous preterm birth (SPTB)**	**After vs before**	1.135 (0.881, 1.436)	0.973 (0.780, 1.185)	0.847 (0.677, 1.059)	1.136 (0.865, 1.492)	0.956 (0.787, 1.161)	0.834 (0.663, 1.049)
	**p-value for interaction**	-	-	0.549	-	-	0.461
**Induced preterm birth (IPTB)**	**After vs before**	0.865 (0.618, 1.210)	1.129 (0.920, 1.384)	0.816 (0.643, 1.034)	0.839 (0.608, 1.159)	1.112 (0.897, 1.378)	0.810 (0.634, 1.036)
	**p-value for interaction**	-	-	0.147	-	-	0.168
**Small for gestational age (SGA)**	**After vs before**	1.351 (1.142, 1.597)	1.037 (0.913, 1.177)	1.225 (1.019, 1.473)	1.339 (1.085, 1.653)	1.030 (0.923, 1.151)	1.175 (0.993, 1.391)
	**p-value for interaction**	-	-	0.103	-	-	0.140
**C-section**	**After vs before**	1.012 (0.912, 1.124)	1.012 (0.949, 1.079)	1.015 (0.906, 1.137)	1.015 (0.907, 1.135)	1.013 (0.947, 1.084)	1.014 (0.903, 1.138)
	**p-value for interaction**	-	-	0.999	-	-	0.999
**No Breast-feeding**	**After vs before**	1.024 (0.986, 1.064)	0.953 (0.872, 1.042)	1.065 (0.959, 1.184)	1.045 (0.977, 1.118)	0.935 (0.840, 1.040)	1.001 (0.885, 1.132)
	**p-value for interaction**	-	-	0.289	-	-	0.307

[#]RR: cumulative risk ratio, CI: confidence interval
[*]LBW adjusting for: mother's education, age, ethnicity, whether in WIC program, and each zip-code area's total population, health insurance coverage, poverty percentage, urban/rural percentage, race and ethnicity percentage; PTB, SPTB, IPTB, SGA, C-section, breastfeeding adjusting for: mother's age, education, ethnicity, pre-pregnancy BMI, whether in WIC program , and each zip-code area's total population, health insurance coverage, poverty percentage, urban/rural percentage, race and ethnicity percentage
[&]Area A=FEMA individual; FEMA, Federal Emergency Management Agency
